# Comprehensive Review of Synthesis, Structure, and Electrochemical Performance of LiFePO_4_

**DOI:** 10.1002/advs.77203

**Published:** 2026-08-28

**Authors:** Lihui Yin, Yao Jiang, Jun Wang, Bin Li, Feng Liu, Cairong Jiang, Jianwu Wen, Chami N. K. Patabendige, Venkataraman Thangadurai, Jianjun Ma

**Affiliations:** ^1^ School of Materials Science and Engineering Sichuan University of Science and Engineering Zigong Sichuan People's Republic of China; ^2^ Institute For Catalysis and Energy Solutions (ICES) University of South Africa Roodepoort South Africa; ^3^ EaStCHEM School of Chemistry University of St Andrews St Andrews UK; ^4^ State Key Laboratory of Environment‐friendly Energy Materials School of Materials and Chemistry Southwest University of Science and Technology Mianyang Sichuan People's Republic of China; ^5^ Department of Science for Technology Faculty of Technology University of Sri Jayewardenepura Homagama Sri Lanka

**Keywords:** cathode material, cyclability, dopants, lithium iron phosphate, lithium‐ion battery, sources

## Abstract

Driven by the growth of the electric vehicle (EV) market, lithium‐ion batteries have gained more attention. Olivine‐structured lithium iron phosphate (LiFePO_4_, LFP) nanomaterials are the most effective cathode materials for lithium‐ion battery applications. In this paper, we review recent advances in LFP cathodes through a unified crystal structure–synthesis–performance–manufacturing framework. We discuss how precursor chemistry, synthesis routes, morphology control, carbon engineering, and doping strategies govern phase formation, charge transport, and electrochemical behavior. Particular attention is given to Fe^2+^ stabilization, synthesis strategy for specific morphology and performance, and emerging recycling and regeneration technologies. We further highlight opportunities in solid‐state batteries, data‐driven materials discovery, and operation under extreme conditions, with fast charging and high‐tap‐density electrode design. By connecting fundamental materials design with industrial implementation, this review provides a roadmap for translating laboratory innovations into next‐generation LFP technologies for sustainable transportation and grid‐scale energy storage.

## Graphical abstract

Olivine‐structured LiFePO_4_ (LFP) nanomaterials are the most effective cathode materials for lithium‐ion battery applications. Here, we report how precursor chemistry, synthesis routes, morphology control, carbon engineering, and doping strategies govern phase formation, charge transport, and electrochemical behaviour of LFP cathodes. Particular attention is given to Fe^2+^ stabilization, synthesis strategy for specific morphology and performance, and emerging recycling and regeneration technologies. We further highlight opportunities in solid‐state batteries, data‐driven materials discovery, and operation under extreme conditions, with fast charging and high‐tap‐density electrode design. By connecting fundamental materials design with industrial implementation, this review provides a roadmap for translating laboratory innovations into next‐generation LFP technologies for sustainable transportation and grid‐scale energy storage.

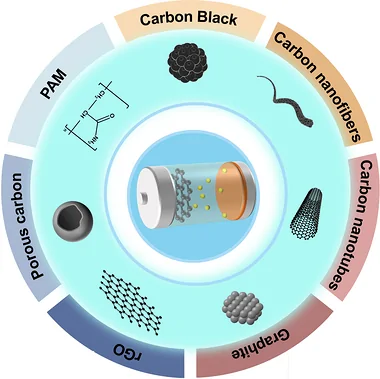

## 1 Introduction

**FIGURE 1:**
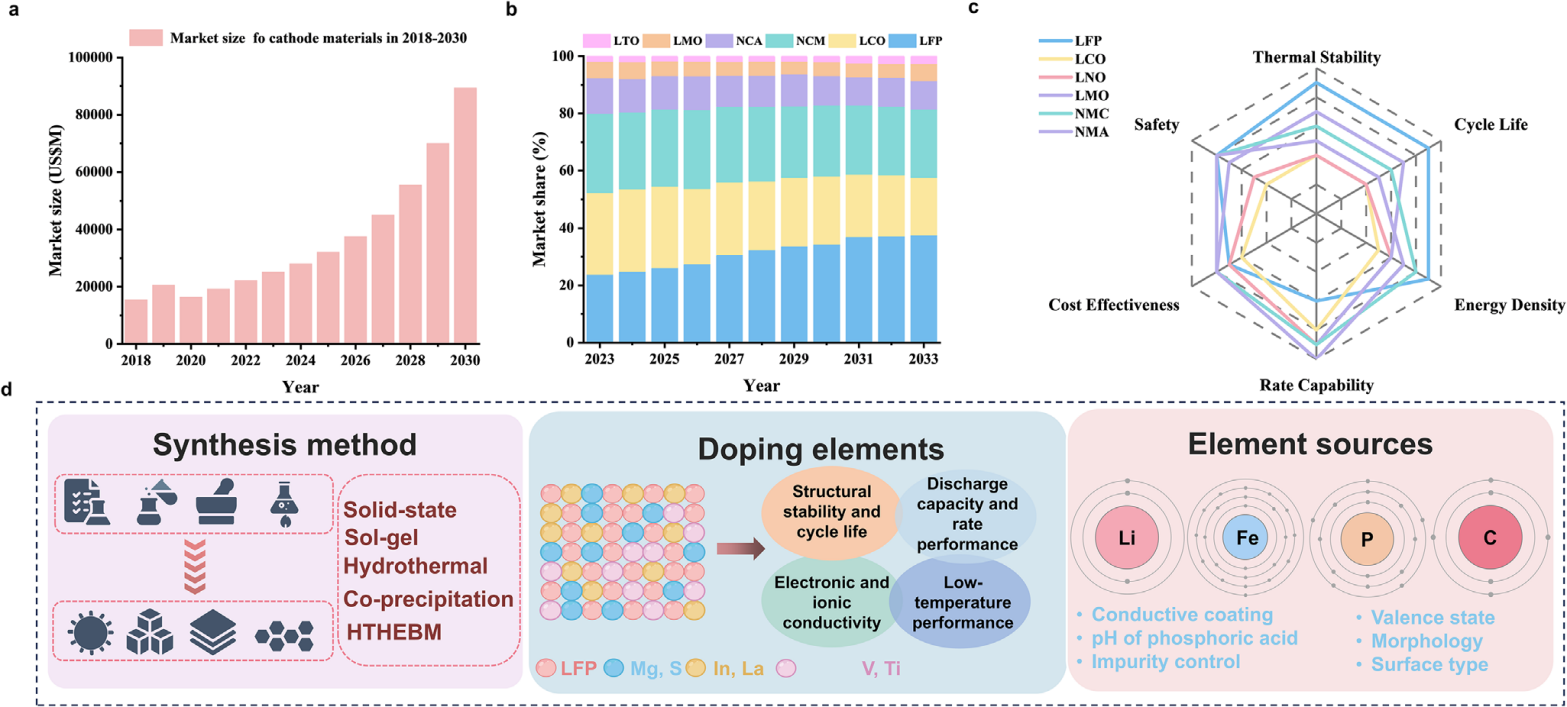
(a) Global market for lithium‐ion batteries (LIBs) [3]; (b) Market percentage of the main cathode materials for LIBs [7]; (c) Changes in the production volume of lithium iron phosphate from 2015 to 2025; (d) Strategy for LFP cathode materials.

Lithium‐ion batteries (LIBs) have attracted significant attention in recent years [1, 2]. The global market is projected to expand from 62.39 USD billion in 2025 to 96.9 USD billion over the next decade, as estimated by Market Research Future (Figure 1a [3]). Growth in the Asia‐Pacific region is significant, driven by government initiatives that promote the adoption of electric vehicles (EVs) [2]. LFP‐based systems are a key technology for large‐scale renewable‐energy integration and the transition toward low‐carbon electricity systems owing to their exceptional safety, long cycle life, low cost, and thermal stability, as compared with lithium cobalt oxide (LiCoO_2_), lithium nickel oxide (LiNiO_2_), lithium manganese oxide (LiMn_2_O_4_, LMO), and ternary compounds like LiNi_x_Co_y_Mn_z_O_2_ (NCM), LiNi_x_Co_y_Al_z_O_2_. LiFePO_4_ (LFP) features a robust olivine structure that provides an exceptional thermal runaway threshold of 210°C –250°C, a theoretical capacity of 170 mAh g^−1^ at 3.4 V, and more than 6000 cycles of lifespan [4, 5]. LFP has the largest share of market production. In 2023, LFP accounted for more than 40% of global electric‐vehicle battery demand by installed capacity [6], and its share will grow steadily (Figure 1b) [7]. This rapid adoption has coincided with a significant reduction in battery costs, with lithium‐ion battery pack prices decreasing by approximately 8% since 2024 to a record low of US$108 kWh^−1^ [8]. The growth is attributed to supportive policies for carbon peaking and carbon neutrality [9]. Although LFP has lower electronic conductivity than LMO or NCM, it remains the dominant cathode material due to its high energy density and cycle life, which can be further enhanced by carbon coating and lattice doping strategies (Figure 1c). Table 1 shows the performance comparison of various cathode materials.

**TABLE 1:** Performance comparison of various cathode materials.

No.	Cathode	Synthesis method	Crystal structure	Particle size (nm)	Energy density	Specific capacity (mAh g^−1^)	Cyclability	Conductivity	Li^+^ diffusivity (cm^2^ s^−1)^	Tap density (g cm^−3^)	Refs.
1	LiFePO_4_	Spray drying	Olivine	100–200	617.8 Wh L^−1^ (10 C)	165.6 (0.1C)	∼100% (5 C, 500 cycles)	2 × 10^−1^ S cm^−1^	—	1.6	[16]
2	LiCoO_2_	Commercial	Layered	—	421.8 Wh kg^−1^ (0.1 C)	196 (0.1 C)	92.4% (0.2 C, 200 cycles)	—	—	—	[17]
3	LiNiO_2_	Sol‐gel	Layered	—	500 Wh kg^−1^	150 (1 C)	90% (1 C, 350 cycles)	—	10^−11^	—	[18]
4	LiMn_2_O_4_	Commercial	Spinel	—	76.76 Wh kg^−1^ (0.2 C)	146 (1 C)	80% (1 C, 300 cycles)	7.2 × 10^−3^ S cm^−1^	—	—	[19]
5	LiNi_0.92_Co_0.06_Mn_0.02_O_2_	Commercial	Layered	—	300 Wh kg^−1^ (1 C)	382.7 (1 C)	91% (1 C, 350 cycles)	—	—	—	[20]
6	LiNi_0.8_Co_0.15_Al_0.05_O_2_	Sol–gel	Layered	150–300	95 Wh kg^−1^	150 (16 C)	72% (10 C, 2000 cycles)	—	—	—	[21]
7	Li_0.99_Na_0.01_FePO_4_	Solvothermal	Olivine	200–500	—	156 (0.1 C)	86.7% (10 C, 500 cycles)	—	1.2325 × 10^−14^	—	[22]
8	LiMn_0.6_Fe_0.4_PO_4_	Hydrothermal	Olivine	—	558.9 Wh kg^−1^	160.7(0.2 C)	93.1% (1 C, 500cycles)	—	—	—	[23]
9	LiFeP_0.9_B_0.1_O_4‐ẟ_/C	Liquid phase reduction	Olivine	200–300	—	155.2 (0.2 C)	99.3% (0.2C, 10 cycles)	—	—	—	[24]
10	LiFePO_3.938_F_0.062_	Solid‐state reaction	Olivine	200–300	—	164.8 (0.1 C)	95.6% (5 C, 500 cycles)	—	2.72 × 10^−12^	1.20	[25]
11	LiFe_0.9_Zn_0.025_Mn_0.075_PO_4_	One‐step hydrothermal	Olivine	—	—	161.8 (0.2 C)	97.5% (0.2 C, 200 cycles)	—	8.63 × 10^−12^	—	[26]
12	Li_0.98_Na_0.02_(Fe_0.65_ Mn_0.35_)_0.97_Mg_0.03_PO_4_/C	Sol–gel	Olivine	—	—	147.7 (0.1C)	96.2% (0.1 C,40 cycles)	—	4.501 × 10^−14^	—	[27]

To date, numerous review articles have summarized the development of LiFePO_4_ cathodes from specific perspectives, including structure and performance [10], recycling of spent LiFePO_4_ [11], synthesis methods [12], carbon‐coating technologies [13], nanostructure design [14], and sustainability considerations [15]. However, many existing reviews treat synthesis methods, morphology regulation, carbon modification, and electrochemical performance as separate topics. As a result, little attention has been given to understanding how synthesis pathways fundamentally govern the interconnected evolution of structure, transport properties, and practical battery performance. Understanding how different synthesis routes govern microstructural evolution and interfacial chemistry is therefore essential not only for maximizing electrochemical performance but also for enabling scalable, economically viable production.

The synthesis strategy for nanoscale LFP effectively overcomes the intrinsic kinetic bottlenecks of its low electronic conductivity and sluggish 1D lithium‐ion diffusion. Some synthesis methods achieve high electrochemical performance by enabling atomic‐level control over specific morphological features and the carbon interface (Figure 1d). These synergistic approaches, coupled with well‐designed precursors and doping, are paving the way for LFP cathodes that challenge the power densities of nickel‐rich alternatives. It is imperative to evaluate how divergent synthesis methodologies precisely control crystal orientation, grain growth, phase purity, and carbon integration.

In this review, we examine recent advances in LFP cathodes through a unified structure–synthesis–performance–manufacturing framework. Particular emphasis is given to the roles of synthesis strategy, precursor chemistry, morphology control, carbon incorporation, and doping engineering in regulating lithium‐ion transport, electronic conductivity, and long‐term cycling stability. By critically evaluating recent developments and connecting fundamental materials design with industrial implementation, we aim to provide a forward‐looking perspective on the development of LFP cathodes for electric mobility and large‐scale energy storage applications.

## 2 Synthesis Strategies and Precursor Chemistry

The electrochemical performance of LiFePO_4_ (LFP) is fundamentally determined by its synthesis history, which governs crystal structure, defect chemistry, particle morphology, and conductive network formation. Although LFP benefits from low cost, high safety, and long cycle life, its intrinsic limitations necessitate careful control of the microstructure through synthesis engineering. As a result, a wide range of synthetic strategies has been developed to regulate nucleation, growth, and carbon integration, thereby enabling high‐performance cathode architectures. However, these approaches differ significantly in their ability to balance electrochemical performance with scalability, cost, and manufacturability. This section discusses the key design principles, precursor chemistry, synthesis routes, and industrial constraints governing LFP production within a unified structure, as well as the performance framework associated with these routes.

### 2.1 Design Principles for LFP Synthesis

**FIGURE 2:**
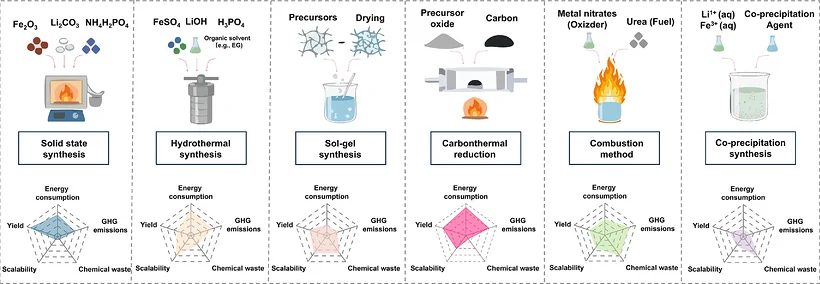
Illustration diagram of synthesis methods for LFP and their characteristics.

While LFP occurs naturally as ore, the high concentration of inherent impurities necessitates synthetic routes to achieve the phase purity required for electrochemical energy storage [28]. Researchers have made efforts to optimize the synthesis of LFP (Figure 2) to improve performance and enable expanded commercial production [29]. The intrinsic limitations include its electronic conductivity in the 10^−9^–10^−10^ S cm^−1^ range and slow Li^+^ diffusivity in the 10^−14^–10^−16^ cm^2^ s^−1^ range. Furthermore, the tap density is 0.8–1.5 g cm^−3^, which is very low. Therefore, the volumetric energy density of LFP batteries is usually lower than that of other battery types [30]. To address these limitations, most research focuses on modulating particle morphology and carbon‐coating architectures to overcome transport constraints. The synthesis method significantly affects the final cation distribution, primary particle size, and surface chemistry, which determine rate capability and cycle life [31].

To date, numerous techniques have been established for synthesizing lithium metal phosphate nanoparticles, such as hydrothermal [32], solvothermal [33], combustion synthesis [34], co‐precipitation [35], sol‐gel [36], template‐assisted [37], spray pyrolysis [38], and solid‐state reaction methods [39]. Among these methods, hydrothermal and solvothermal methods efficiently control crystal orientation and produce uniform nanosized grains through high‐temperature and high‐pressure solution reactions. However, their main limitation is the need for specialized high‐pressure corrosion‐resistant equipment, along with safety concerns and increased chemical waste [32]. The sol‐gel method enables molecular‐level mixing and high compositional uniformity, making it suitable for time‐consuming applications, but it is prone to shrinkage and cracking during drying [36]. The co‐precipitation method provides a simple, low‐cost route for tuning composition and morphology by controlling reaction parameters [40]. However, the precipitation step is sensitive to impurities and compositional inhomogeneity, and subsequent washing and filtration may be energy‐intensive and introduce pollution [41]. The combustion synthesis method offers ultra‐fast kinetics and can achieve phase formation within seconds. These rapidly formed nanostructures effectively mitigate sluggish Li^+^ diffusion and poor electronic conductivity (10^−10^ S cm^−1^), enabling high‐throughput scalability [42]. However, this process is associated with higher greenhouse gas (GHG) emissions. The template‐assisted method allows precise microstructural control, but template removal and limited process flexibility restrict scalability [37]. Spray drying has the advantage of continuous large‐scale production, yielding particles with a regular shape and uniform size, but it requires more advanced equipment and higher energy consumption [30].

Although sophisticated solution‐based techniques have been developed, solid‐state reaction remains the dominant industrial LFP production due to its scalability and process robustness. Additionally, it facilitates the Fe^3+^→Fe^2+^ reduction and in situ carbon formation when combined with carbothermal reduction [43]. While sol‐gel and hydrothermal methods provide superior control over microstructure and morphology, their commercial viability is often limited by process complexity, low throughput, and challenges associated with scale‐up and yield [44].

The critical precursors, such as lithium and iron precursors, phosphate and carbon sources, are required for the synthesis of LFP cathode materials. The precursors are deemed to affect LFP performance. Ore resources and the purification process also affect cathode production [45]. The Li and Fe precursors are the core raw materials that determine performance, and their purity and stoichiometric ratios must be strictly controlled. The C source controls the dosage during selection and serves as both a reducing agent and a conductive coating layer during sintering when the solid‐state reaction method is used. In this section, we will discuss the effects of various Li and Fe precursors and C sources on the performance of LFP batteries.

### 2.2 Li Precursors

Lithium precursors are essential components in the preparation of lithium iron phosphate. The morphology of LFP materials and the electrochemical performance of batteries are affected by the precursors. Common Li precursors include lithium carbonate (Li_2_CO_3_), lithium hydroxide monohydrate (LiOH·H_2_O) [46], lithium fluoride (LiF) [47], and lithium acetate (C_2_H_3_LiO_2_, CH_3_COOLi·2H_2_O) [48].

Li_2_CO_3_ and LiOH·H_2_O are widely used as lithium precursors for LFP [49]. Li_2_CO_3_ is the dominant industrial source of lithium because of its comparatively low cost. Its cost is 30%–50% lower than battery‐grade LiOH on a molar lithium basis. It also has high chemical stability and a well‐established supply chain derived from brines or rocks [50]. Recovered Li_2_CO_3_ was used as the lithium precursor for LiFePO_4_, and the battery exhibited good performance [51]. However, Li_2_CO_3_ exhibits relatively sluggish decomposition, which requires a calcination temperature between 650°C and 800°C during the preparation of LiFePO_4_. This excessive calcination temperature often accelerates particle coarsening. For example, sintering Li_2_CO_3_ as a lithium precursor at 800°C results in particles that are coarsened to >5 µm. An alternative route using lithium fluoride (LiF) as a lithium precursor was developed. The cell with LiF@600°C, with fine particles of 500 nm, exhibits a specific capacity of 151 mAh g^−1^ at 1/15 C [47]. From a kinetic perspective, the lithium‐ion diffusion path is shorter in smaller particles. This structural advantage is directly converted into excellent electrochemical performance. The significant size difference highlights the unique benefits of LiF precursors in suppressing particle overgrowth. Another issue associated with the use of Li_2_CO_3_ is that incomplete decomposition can produce residual Li_2_CO_3_ and the Li_3_PO_4_ impurity phase. These materials are electronically insulating and consequently increase the interfacial resistance [52].

Lithium acetate is another Li precursor used by predecessors [48, 53]. For example, an olivine‐structured LFP was successfully synthesized from lithium acetate (C_2_H_3_LiO_2_), ferrous oxalate, diammonium phosphate, and citric acid as raw materials using the low‐temperature solid‐phase coordination method combined with microwave heating [53]. The morphology and electrochemical properties indicated that the sample without citric acid had a wide particle size range of 200–800 nm. With increasing citric acid content, the particle size decreases to 80–150 nm, 40–50 nm, and 10–20 nm for the samples containing 1.25, 2.5, and 3.75 mmol citric acid, respectively. The results show that the best performance is obtained with a sample containing 2.5 mmol citric acid, achieving a capacity of 125 mAh g^−1^ at 0.5 C and stable cycling.

By contrast, LiOH has been used to synthesize high‐performance LFP composites due to its higher chemical reactivity and lower activation energy for lithiation. Some researchers have prepared nanostructured mesoporous LFP/C composites by spray‐drying‐assisted solid‐state reaction using LiOH as raw materials [46]. In this method, the traditional calcination process is replaced by a micro‐reduction atmosphere produced by embedding rice husk carbon in a box furnace, which has multiple functions. This atmosphere is conducive to the formation of pure‐phase LFP and to the reduction of Fe^3+^ to Fe^2+^. At the same time, rice husk carbon and glucose synergistically form a crystalline carbon network, yielding high‐performance LFP/C materials. Electrochemical tests show that the LFP/C cathode material has a specific capacity of 158 mAh g^−1^ at 0.2 C and remains 128 mAh g^−1^ at 10 C. It can be seen that LiOH‐derived LFP often demonstrates improved rate capability and lower polarization under high‐current operation, which may result from enhanced lithium diffusivity that suppresses excessive grain growth and facilitates the formation of nanoscale particles with shorter lithium‐ion diffusion pathways and larger electrode–electrolyte interfacial areas. However, LiOH is hygroscopic and readily reacts with atmospheric CO_2_ to form Li_2_CO_3_ at the surface. In this case, a non‐stoichiometric precursor is introduced, which increases the likelihood of lithium‐deficient impurity phases. Furthermore, lithium volatilization at elevated temperature can produce Fe_2_P or Fe‐rich secondary phases if the Li/Fe ratio is not well controlled. The generation of large volumes of corrosive wastewater is another critical consideration in large‐scale production and has prevented its use in industrial cathode manufacturing.

**FIGURE 3:**
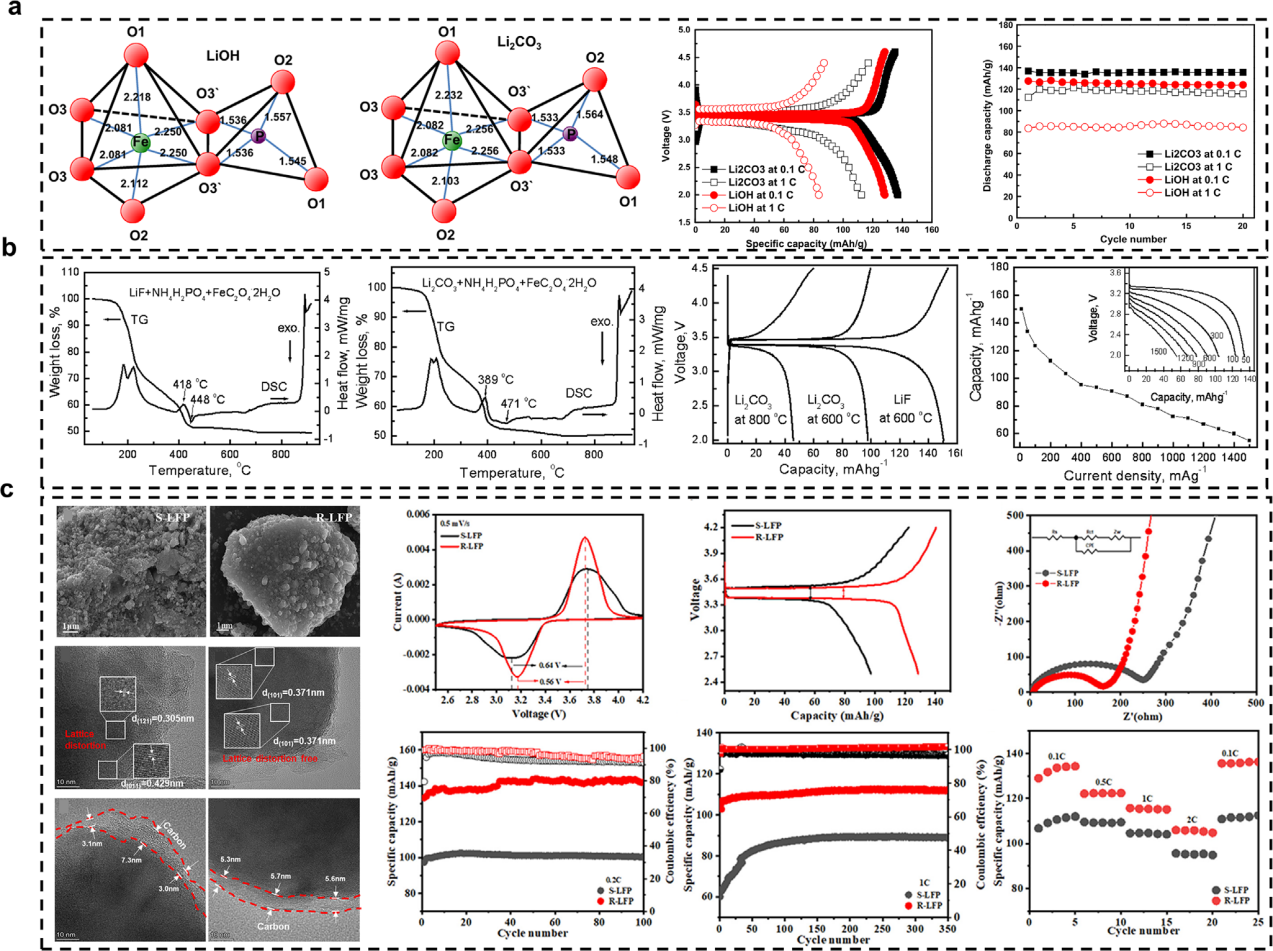
(a) Bond distance and electrochemical performance of LFP prepared by multifunctional lithium source, reproduced with permission [54]. Copyright 2013, Elsevier; (b) Sintering mechanism of various lithium sources, and their performance, reproduced with permission [55]. Copyright 2004, Elsevier; (c) Effect of morphology of lithium sources on electrochemical performance, reproduced with permission [56]. Copyright 2025, AIP publishing.

Precursor selection is critical to local coordination and electrochemical performance of LFP. Figure 3a shows that Li_2_CO_3_ precursors induce lattice distortions, with shorter Li─O and P─O_3_ bonds and longer Fe‐O_1_ distances [54]. These shifts enhance P─O covalency, optimizing the Fe^2+^/Fe^3+^ redox potential through the inductive effect. Li_2_CO_3_‐derived frameworks facilitate zigzag transport by reducing jump distances between LiO_6_ octahedra via PO_4_ junctions. Therefore, a higher capacity of 136.9 mAh g^−1^ at 0.1 C was achieved, compared to 127.5 mAh g^−1^ for the LiOH‐derived precursor at the same rate. Figure 3b shows that LiF‐based routes initially involve dehydration between 183°C and 221°C, followed by phase formation between 390°C and 460°C. Li_2_CO_3_ decomposes earlier but requires a higher crystallization temperature of around 471°C [55]. The thermal treatment of LiF at 750°C promotes nucleation and grain growth, which are essential for high‐performance cathodes. Furthermore, these principles extend to the regeneration of spent LFP. The regenerated LFP performs much better than the spent one (Figure 3c) [56]. Multifunctional lithium sources can simultaneously replenish lattice vacancies and generate in situ carbon coatings. This encapsulation creates a reducing atmosphere suitable for the reduction of Fe^3+^ to Fe^2+^. In this case, electronic conductivity was enhanced, and particle pulverization occurred during extended cycling.

### 2.3 Fe Precursors

The selection of an iron precursor is a decisive factor in determining the structural integrity, morphological evolution, and electrochemical efficiency of LFP cathodes. To date, many iron sources have been used in their synthesis, such as FePO_4_·xH_2_O [57], Fe_2_O_3_ [58, 59], Fe(OH)_3_ [60], and FeSO_4_ [61]. These precursors are primarily categorized by their oxidation state: ferrous (Fe^2+^) or ferric (Fe^3+^). Each requires distinct thermodynamic pathways to achieve the desired olivine structure. Ferrous sources like FeC_2_O_4_·2H_2_O (ferrous oxalate) are highly regarded for solid‐state synthesis because their decomposition releases CO and CO_2_, which naturally creates a reductive environment that prevents the formation of electrochemically inactive impurities [62]. Similarly, FeSO_4_·7H_2_O [63] and Fe(NH_4_)_2_(SO_4_)_2_·6H_2_O [64] offer high solubility and are staples in hydrothermal methods. They are highly soluble in water, which allows for molecular‐level mixing with lithium and phosphate sources. This often leads to LFPs with exceptional crystallinity and specific exposure of crystal facets (e.g., the [010] plane). Compared with FeSO_4_, FeC_2_O_4_ is more expensive and generates CO‐containing off‐gases during decomposition. In industrial processing, additional gas‐treatment systems are usually required. Sometimes the presence of sulfur in FeSO_4_ has both advantages and disadvantages. When sulfate ions are not rigorously washed away, they can remain as impurities that trigger side reactions with the electrolyte, leading to gas evolution and cell swelling during cycling. The purity of FeSO_4_ was found to strongly influence the morphology of the precursor FePO_4_ but to have little effect on the morphology of the resulting FPC material. In contrast, electrochemical performance was highly sensitive to the purity of FeSO_4_. Both cycling stability and low‐temperature discharge performance deteriorated markedly as the purity decreased. At −20°C and 0.5 C, the discharge specific capacity decreased from 85.3 mAh g^−^^1^ for the high‐purity sample to 50.1 mAh g^−^^1^ for the low‐purity sample, with the corresponding capacity retention declining from 53.3% to 31.9% [61]. The performance degradation might be attributed to the Ti impurity in low‐purity FeSO_4_. The content of Ti content is 189 and 8200 ppm. Although it is uncommon to have a high Ti impurity in FeSO_4_, the results in this study show that the Ti impurity is a major factor [61]. This impurity induces lattice distortion and blocks the Li^+^ channel. In this case, the Li^+^ diffusion coefficient (D‐Li^+^) decreases from 10^−13^ to 10^−15^ K^−1^.

Conversely, ferric (Fe^3+^) precursors, such as Fe_2_O_3_ (Hematite) and Fe(OH)_3_, are favored for industrial‐scale production due to their abundance and low cost. However, these materials require carbothermal reduction, a process where carbon additives simultaneously reduce iron to the divalent state and form a conductive surface coating. It was reported that LFP was successfully prepared with optimal lithium content by thermal reduction using inexpensive, readily available Fe_2_O_3_ as the iron precursor [65]. In the half cell (2.0–4.2 V), the reversible capacity is 153.8 mAh g^−1^. The material exhibits better C‐rate and cycle life in a flexible packaging full battery. In this study, they propose a low‐cost synthesis route that offers a viable pathway for the large‐scale application of olivine‐structured LFP in practical full batteries. Although Fe_2_O_3_ is an economically attractive precursor, its high sintering temperature promotes undesirable grain growth and therefore hinders lithium‐ion transport kinetics. The choice of iron source also governs the resulting particle size and the quality of the carbon‐iron phosphate interface. For example, FeC_2_O_4_ decomposes during use and tends to produce smaller, more porous particles that shorten the diffusion paths for lithium ions.

The morphology and properties of LFP are also affected by different iron precursors. A systematic study was conducted to compare three different iron sources, Fe_2_O_3_, Fe(OH)_3_, and FeSO_4_, for the preparation of LFP compounds [60] by solid‐state carbon thermal reduction. Electron microscopy analysis showed that although all samples exhibited a porous morphology, their porosity differed significantly depending on the iron source. The samples prepared using the iron source at 700°C were used for the performance test. Among the investigated precursors, the sample synthesized using Fe(OH)_3_ as the iron source exhibited the best electrochemical performance, delivering a discharge capacity of 115 mAh g^−1^ at 0.2 C, approximately 20% higher than that obtained using Fe_2_O_3_. This enhancement was attributed to a higher pore fraction.

**FIGURE 4:**
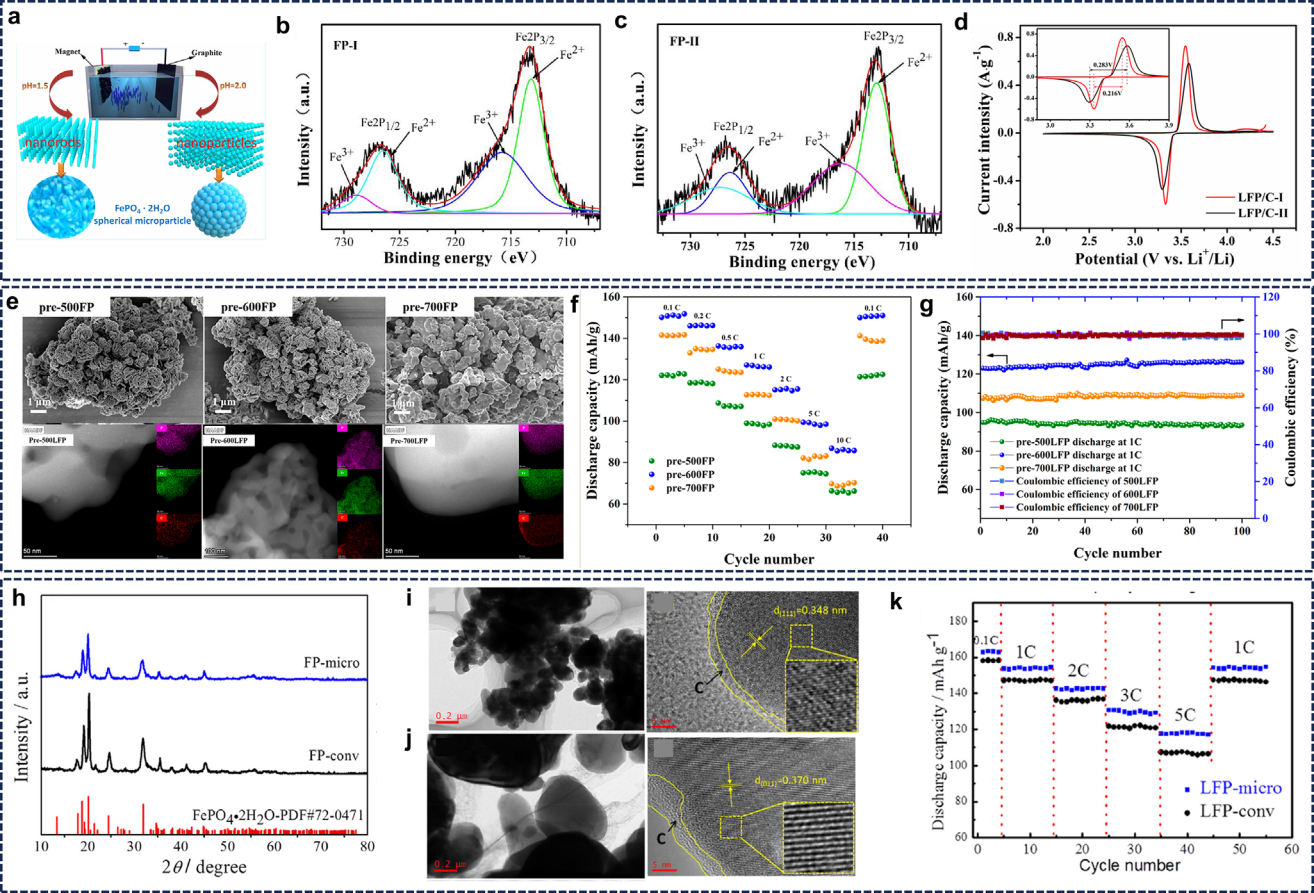
Iron precursors for LFP. (a–d) Iron precursors with different pH values, reproduced with permission [68]. Copyright 2023, American Chemical Society; (e–g) Influence of crystallinity on the electrochemical performance of pretreated LFP at 500°C (pre‐500LFP), 600°C (pre‐600LFP), and 700°C (pre‐700LFP), reproduced with permission [67]. Copyright 2022, American Chemical Society; (h–k) Effects of synthesis strategy on the electrochemical performance, reproduced with permission [69]. Copyright 2019, Elsevier.

The crystallinity and solubility of the iron source are other important factors. High‐crystallinity iron sources, such as anhydrous FePO_4_, often yield superior phase purity and symmetrical particle‐size distributions because it inherently contains the correct Fe:P stoichiometric ratio. When carbon is coated on the surface, it forms a uniform layer. The homogeneous carbon coating and improved electronic and ionic transport resulted in a higher discharge capacity of 146.7 mAh g^−1^ at 1 C than that of FePO_4_·2H_2_O (135.1 mAh g^−1^) [66]. The use of FePO_4_ minimizes local stoichiometric fluctuations and improves batch‐to‐batch consistency, which is critical for large‐scale battery manufacturing. In contrast, the dehydration of lower‐crystallinity hydrous sources can lead to uneven carbon distribution, increasing charge‐transfer resistance [67]. Peng et al. adjusted the pH of the FePO_4_ precursor, prepared via anodic flocculation in a phosphoric acid/hydrogen peroxide solution using iron‐filing waste, from 2.5 to 1.0 (Figure 4a–d) [68]. Their results showed that decreasing the solution pH from 2.0 to 1.5 increased the Fe^2+^ content from 20.3% to 30.5%, indicating improved stabilization of ferrous species and suppression of Fe^2+^ oxidation during precursor formation. The slightly improved electrochemical kinetics, as indicated by CV curves (Figure 4d), could be due to the smaller particle size of the sample at pH = 1.5 compared with pH = 2.0.

Crystallinity is another significant factor that affects battery performance. The LFP samples treated at 500°C, 600°C, and 700°C are denoted pre‐500LFP, pre‐600LFP, and pre‐700LFP, respectively. Among them, pre‐600LFP exhibited the best electrochemical performance, delivering an initial discharge capacity of 130.0 mAh g^−1^ with a capacity retention of 102.9% after 100 cycles, suggesting progressive activation during early cycling. In contrast, pre‐500LFP and pre‐700LFP displayed lower capacities (Figure 4e–g). The superior behavior of pre‐600LFP is attributed to its optimal crystallinity and a fine‐pored precursor structure, which enables smaller particles, uniform carbon coating, shorter Li^+^ diffusion paths, and improved conductivity. Synthesis routes yield significantly different final electrochemical performance. Hu et al. presented the influence of particle size on rate performance using the same FePO_4_·2H_2_O type but with different synthesis routes (Figure 4h–k). The LFP presents a particle size distribution from 0.1 to 0.3 µm prepared by a microreaction‐assisted synthesis method, compared with 0.24–1.07 µm of that prepared by the conventional precipitation method (Figure 4h). Consequently, the discharge capacities of the former and latter are 117.6 mAh g^−1^ and 106.9 mAh g^−1^ at 5 C, respectively [69]. Although Fe(NO_3_)_3_‐derived LFP exhibits excellent electrochemical performance at the laboratory scale, this route remains less industrially attractive for two reasons [70]. First, a reduction process is usually required during heat treatment to reduce ferric (Fe^3+^) to ferrous (Fe^2+^). Second, nitrate decomposition releases substantial quantities of NO_x_ gases. This not only raises environmental and safety concerns but also complicates industrial‐scale operations and increases processing costs.

### 2.4 Ratio of the Elements

**FIGURE 5:**
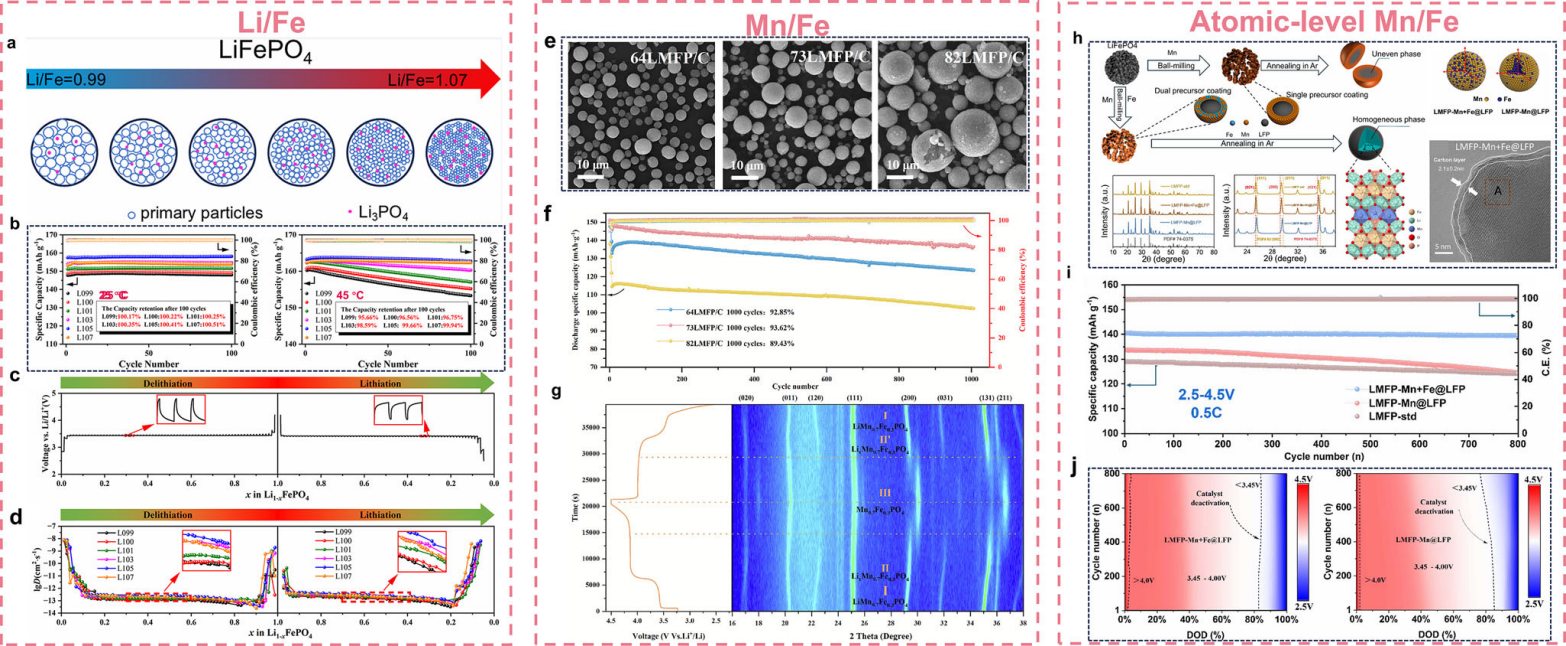
(a–d) Electrochemical performance of LFP batteries with various Li/P ratios, reproduced with permission [71]. Copyright 2025, American Chemical Society; (e–g) Electrochemical performance of the LiMn_y_Fe_1‐y_PO_4_/C battery with various Mn/Fe ratios, reproduced with permissoin [72]. Copyright 2026, Elsevier; (h–j) Dual precursor coating method for homogeneous Mn‐Fe distribution at the atomic scale, reproduced with permission [73]. Copyright 2026, The Royal Society of Chemistry.

Not only the precursor but also the ratio of these elements influences LFP battery performance. Figure 5a–d shows that lower‐Li/P‐stoichiometry samples maintain 97.8% Coulombic efficiency and stabilize the host structure against long‐term degradation. It is critical to reduce defect‐induced fragmentation and optimize cation ratios to achieve high‐performance, long‐life polyanionic cathodes [71]. Recent advances in olivine‐structured cathode materials show that precise stoichiometric control of the Mn/P and Li/P ratios is essential to balance energy density with structural longevity. A high Mn: P ratio promotes primary nanoparticle growth. However, there is an optimized concentration. A high concentration induces lattice stress, leading to fragmentation of secondary spherical aggregates and ultimately increasing capacity loss per cycle (Figure 5e–g) [72]. A dual‐precursor coating strategy was designed to overcome the kinetic limitations of lithium manganese iron phosphate (LMFP) by precisely engineering its atomic‐level lattice. By employing a mechanochemical method with two‐step carbon coating‐thermal annealing, manganese and iron sources were integrated into a lithium iron phosphate (LFP) template (Figure 5h–j) [73].

### 2.5 C Sources

**FIGURE 6:**
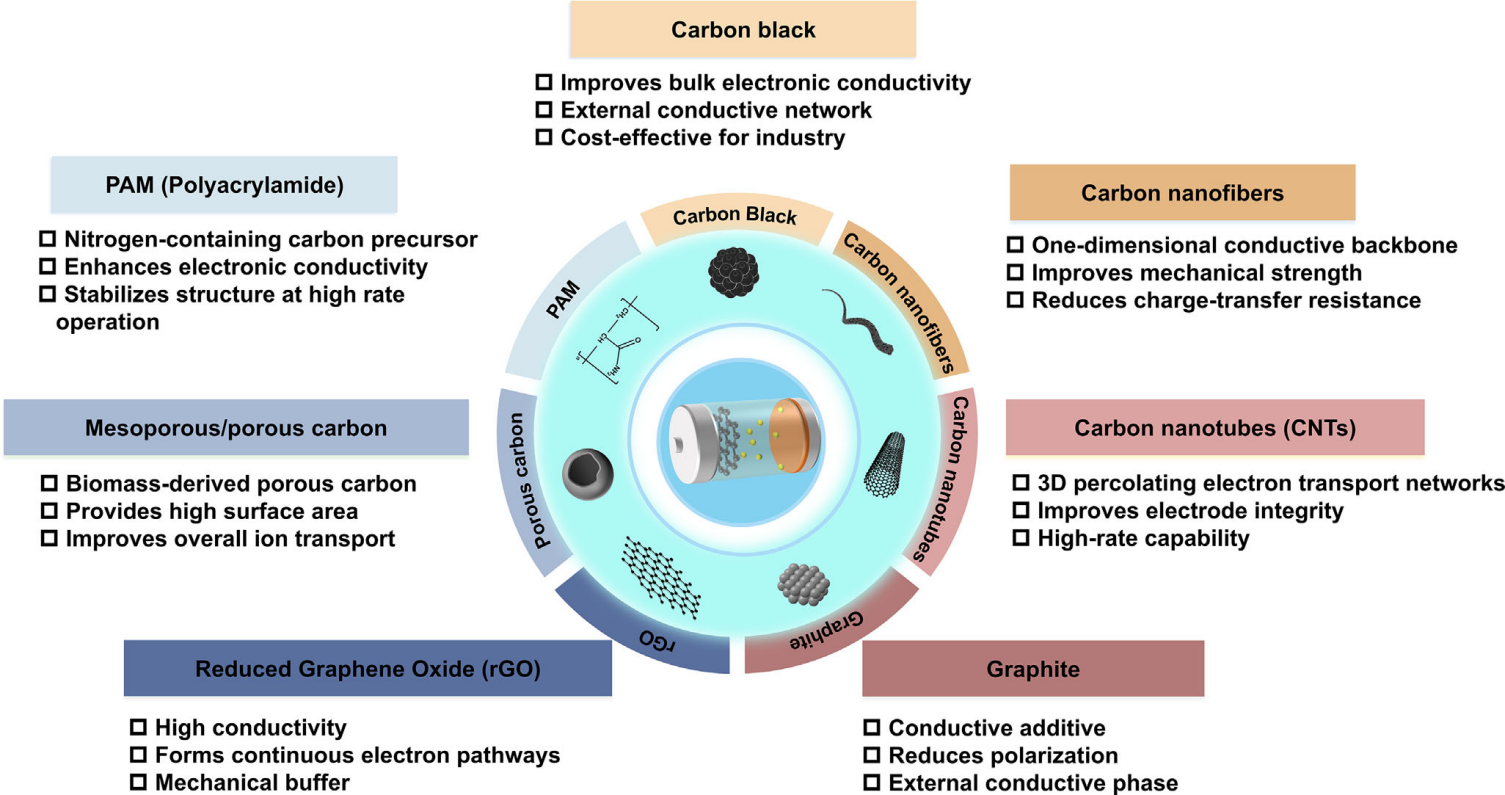
Illustration diagram of carbon material for LFP‐based batteries.

Carbon coating is widely employed to enhance the intrinsic low electronic conductivity of LFP. The electrochemical performance is highly sensitive to carbon precursor, coating thickness, and deposition methodology [74]. For example, the surface coating of N‐doped carbon is 5.2 nm, thinner than the 5.8 nm of pure carbon, thereby shortening the Li^+^ diffusion pathway [75]. Figure 6 summarizes the functions of carbon sources in LFP cathodes. Depending on their roles in enhancing conductivity and regulating structure, carbon precursors can be broadly classified into conventional conductive carbon coatings, nitrogen‐doped carbon coatings, multidimensional conductive networks, and emerging carbon precursors for morphology control and sustainable synthesis.

It can be seen that carbon nanotubes, carbon nanofibers, graphite, and graphene have been widely used as carbon sources for LFP‐based batteries. The batteries benefit from their conductivity, 3D network, and the creation of conductive paths. There are many kinds of carbon sources used in the preparation of lithium iron phosphate, including adipic acid [76], natural sawdust [77], Tween60‐Span60 composite [78], polyethylene glycol (PEG) [79], and others. Carbon fibers were used as the carbon source for LFP‐based batteries, contributing to low resistance and a low degradation rate during cycling [80]. These precursors range from simple carbohydrates to complex synthetic polymers and form a conductive carbonaceous network during controlled pyrolysis in an inert atmosphere at 600°C–800°C.

#### 2.5.1 Conventional Conductive Carbon Coating

Small‐molecule carbohydrates, such as glucose and sucrose, are cost‐effective and highly soluble precursors that facilitate the formation of uniform nanoscale carbon coatings. A glucose‐based carbothermal reduction route has been reported for the synthesis of LFP/C composites [81]. In this approach, glucose‐derived carbon was obtained at 600°C for 24 h. The resulting material delivered a specific capacity of 121 mAh g^−1^ at 1 C and retained 119 mAh g^−1^ after 30 cycles. Glucose has also been used as a multifunctional agent in the recycling process of LiFePO_4_. A 1.6‐nm‐thick carbon layer was coated onto the regenerated black mass. Glucose was a reductant in the hydrothermal process. Specifically, it is a dispersant and morphology‐regulating agent and promotes favorable particle rearrangement and structural homogenization during regeneration [82]. In contrast, synthetic polymers, like PVA [16] and PEG [83], offer dual‐functional roles as both carbon sources and structural templates. To enhance the volumetric energy density of LFP/C, xylitol–PVA has been employed as a composite carbon source [16]. During synthesis, molten xylitol coats FePO_4_ particles, whereas the PVA hydrogel functions as both a dispersant and network‐forming agent. The results showed that the sample containing a mixture of xylitol and PVA delivered 128.7 mAh g^−1^ at 10 C at room temperature and retained nearly 100% of its capacity after 500 cycles at 5 C. The good performance may be attributed to an electronic conductivity of 2 × 10^−1^ S cm^−1^ and a tap density of 1.6 g cm^−3^ in a 2–10 µm microspheres carbon framework core–shell structure. PVA has been proven to be an effective morphology‐control agent in spray‐drying scale‐up, promoting the transformation of solid spherical particles into hollow structures. The battery using PVA‐derived carbon exhibits 91% capacity retention at 1 C after 750 cycles [84].

Polyethylene glycol (PEG) has also been used as a multifunctional additive acting as a carbon source, dispersant, and reducing agent [83]. When combined with carbon aerogel, PEG promotes the formation of a unique mortar‐like conductive architecture and improves the uniformity of the carbon coating. The composite delivered reversible capacities of 163.4 mAh g^−1^ and 115.4 mAh g^−1^ at 0.2 C and 15 C with 99.3% capacity retention after 300 cycles at 5 C.

#### 2.5.2 Heteroatom‐Doped Carbon Coatings

**Heteroatom‐doped carbon coatings,** such as PAM and PAN, enable in situ nitrogen doping during pyrolysis, introducing pyridinic and pyrrolic species that enhance electronic conductivity. It has been reported that the LFP battery with PAM‐derived carbon achieved stable high‐rate performance of 78.9 mAh g^−1^ at 30 C with 98% retention after 100 cycles [85]. Similarly, a PAN‐derived carbon‐coated LFP battery delivered 164.1 mAh g^−1^ at 0.1 C and 49.0 mAh g^−1^ at 50 C [86]. These improvements are attributed to modifications to the electronic band structure of the carbon coating and reduced charge‐transfer resistance (R_ct_), making nitrogen‐doped carbon coating particularly attractive for high‐rate performance in power‐dense applications.

#### 2.5.3 Multidimensional Conductive Networks

**FIGURE 7:**
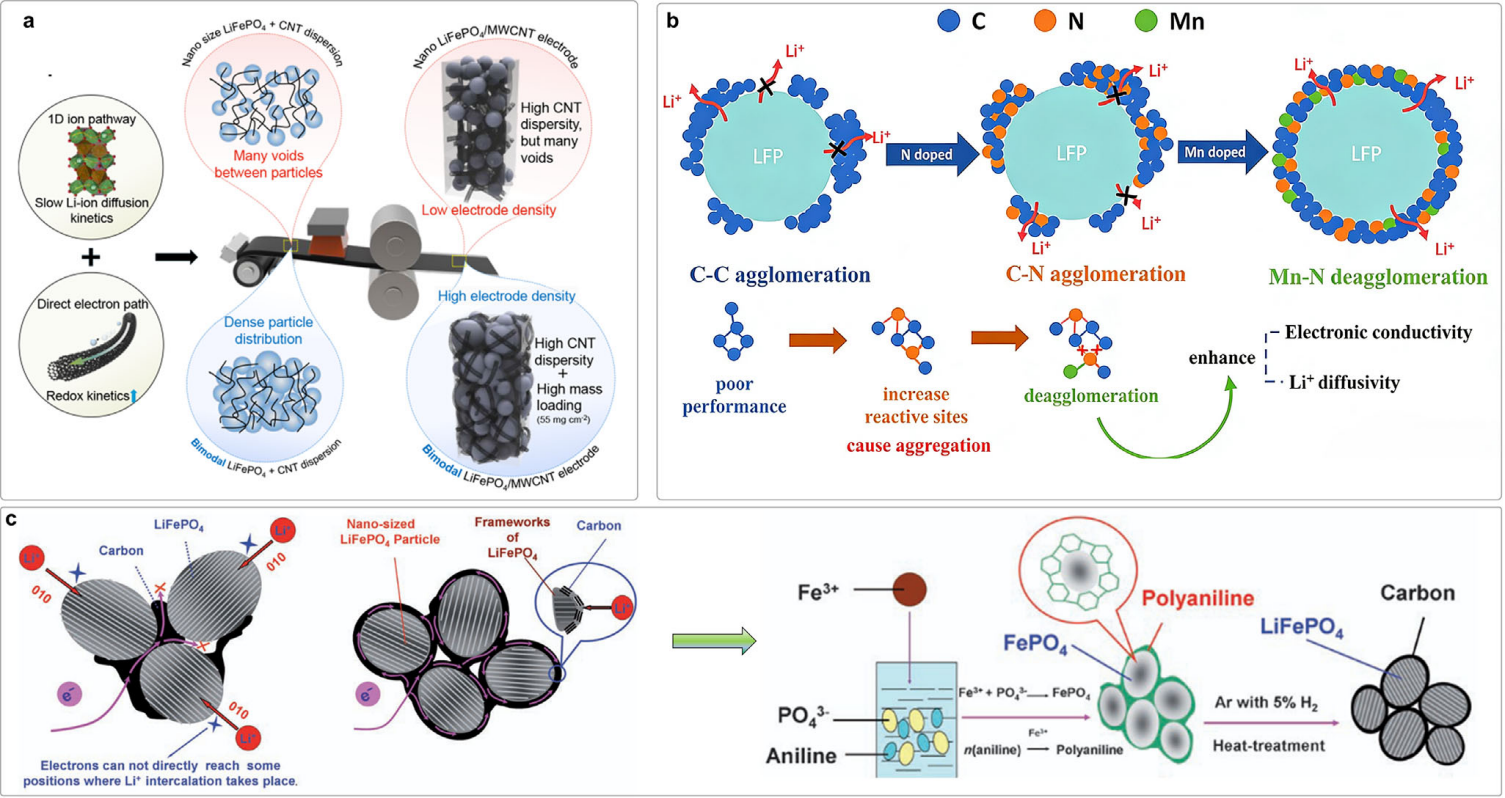
(a) Illustration diagram of a multiwalled carbon nanotube for the LFP thick electrode fabrication, reproduced with permission [87]. Copyright 2025, American Chemical Society; (b) illustration diagram of N‐Mn co‐doped carbon layer coating on LFP, reproduced with permission [75]. Copyright 2024, American Chemical Society; (c) Schematic illustration of LFP/carbon with a core–shell structure using the in situ polymerization method, reproduced with permission [88]. Copyright 2008, Wiley‐VCH.

Multiwalled carbon nanotubes (MWCNTs) are another carbon source for LFP as they facilitate long‐range electron transport throughout the electrode due to their multidimensional conductive frameworks. Electrodes containing 5 wt.% MWCNTs exhibited reduced redox polarization and enhanced electrochemical performance. A high capacity of 7.27 mAh cm^−2^ at 0.3 C is achieved, with 80.74% retention after 90 cycles (Figure 7a) [87]. To further optimize charge transport, a synergistic N‐Mn co‐doping method has been developed to regulate carbon coating uniformity and suppress C–N aggregation (Figure 7b). The as‐prepared battery delivers 119 mAh g^−^^1^ at 10 C and maintains 88% capacity retention over 600 cycles at 5 C. These interconnected conductive frameworks enhance electron and Li^+^ transport, highlighting the importance of integrated conductive networks in high‐rate LFP cathodes [75]. An in situ polymerization–confinement strategy has also been designed to construct LFP/carbon composites with well‐defined core–shell architectures, in which nanoscale LFP (≈20–40 nm) is uniformly coated by an ultrathin (1–2 nm) semi‐graphitic carbon layer (Figure 7c) [88]. This conformal coating enhances interfacial charge transfer while preserving structural integrity.

#### 2.5.4 Novel Carbon Precursor

The diversification of precursors into surfactants and biomass reflects a shift toward morphological precision and environmental sustainability. Amphiphilic molecules like Tween60 and Span60 function as surface‐active agents in hydrothermal synthesis, directing the growth of LFP into optimized geometries, including nanoplates and microspheres. The Tween60‐Span60 composite refers to a mixture in which the raw material is first combined with Tween60, then further mixed with Span60. The weight ratio of Tween60 to Span60 in this composition is 3 [78]. The LFP/C composite material prepared using Tween60 and Span60 as carbon sources is referred to as LFP/C‐TS. However, the order in which Span60 and Tween60 are added is reversed in the case of the Span60‐Tween60 composite. The LFP/C composite material prepared using Span60‐Tween60 as a carbon source is referred to as LFP/C‐ST. The Tween60‐Span60 composite exhibits strong steric hindrance, which increases surface energy and reactivity. As a result, compared to LFP/C‐ST, LFP/C‐TS exhibits a more uniform particle distribution and significantly smaller particle sizes. LFP/C‐TS material thus exhibits a high capacity of 166–130.1 mAh g^−1^ at a rate of 0.1 C in a wide temperature range from 25°C to −20°C, and the capacity remains stable after hundreds of cycles.

Biomass‐derived carbon sources, such as natural sawdust, have attracted growing interest because of their low cost, renewability, and circular economy mandates. These sustainable novel precursors provide an environmentally benign pathway for carbon coating and may contribute to the development of low‐carbon battery manufacturing. By selecting precursors based on their specific pyrolysis chemistry (e.g., whether for nitrogen doping, morphology control, or sustainability), researchers can tailor the LFP interface to meet the rigorous energy and power density benchmarks of next‐generation storage systems.

Table 2 lists a comparison of lithium, iron, and carbon precursors used in LFP synthesis. It can be seen that variations in source selection influence crystallinity, particle‐size distribution, and the nature of the carbon coating, which determine electrochemical behavior. In industrial applications, it is necessary to precisely control synthesis parameters, such as temperature, reaction time, and atmosphere, to ensure complete reaction at high sintering temperatures. Regarding Li sources, lithium carbonate (Li_2_CO_3_) remains the dominant industrial choice, given the requirement of complete reaction at high sintering temperatures. Lithium hydroxide (LiOH·H_2_O) is highly reactive and can react with iron and phosphorus sources at low temperatures, which is beneficial for controlling the reaction process and grain growth. Among a variety of Fe sources, ferrous oxalate (FeC_2_O_4_·2H_2_O) is one of the commonly used iron sources. Ferrous oxalate is highly reactive and can be stored and used under an inert atmosphere; it can react with lithium and phosphorus sources at lower temperatures to form lithium iron phosphate. Ferrous oxalate releases carbon dioxide and water during the reaction, which helps promote the solid‐state reaction. FePO_4_‐derived LFP has been reported to deliver a higher discharge capacity of 141.4 mAh g^−1^ than its hydrated counterpart of FePO_4_·2H_2_O, while maintaining stable cycling over 200 cycles. This might be attributed to improved crystallinity, more uniform carbon coatings, and a controlled particle‐size distribution [66]. In contrast, iron oxide (Fe_2_O_3_) offers a cost‐effective precursor that can be reduced from Fe^3+^ to Fe^2+^ during synthesis in the presence of carbon sources. Carbon improves LFP performance mainly by increasing its electronic conductivity. The carbon‐coated cathode's interfacial resistance is reduced. The other advantage is that carbon helps limit particle growth, maintain structural stability, and improve contact with the electrolyte, which together support faster lithium‐ion transport and stable cycling.

**TABLE 2:** Performance comparison of different Li, Fe, and C sources used in the preparation of lithium iron phosphate by the solid‐phase method.

No.	Li source	Fe source	C source	Particle size	Synthesis method	Sintering condition	Specific capacity	Cycling stability	Refs.
1	Li_2_CO_3_	FePO_4_	Glucose, polyethylene glycol/carbon nanotubes	—	High‐temperature solid‐phase	750°C for 5 h	113.5 mAh g^−1^ (15 C)	96.6% (15 C, 300 cycles)	[79]
2	Li_2_CO_3_	FePO_4_	Glucose	100‐500 nm	Liquid phase crystallization+ high‐temperature solid phase	700°C for 6 h	159.29 mAh g^−1^ (0.1 C)	—	[89]
3	Li_2_CO_3_	FePO_4_	Xylitol‐polyvinyl alcohol	100‐200 nm	ball‐milling, spray drying and carbothermal reduction	750°C for 4 h	165.6 mAh g^−1^ (0.2 C)	∼100% (5 C/5 C, 500 cycles)	[16]
4	Li_2_CO_3_	FePO_4_	Polyethylene glycol	5–30 nm	Ball milling‐spray drying‐carbothermal reduction	720°C for 5 h	163.4 mAh g^−1^ (0.2 C)	99.3% (5 C/5 C, 300 cycles)	[83]
5	Li_2_CO_3_	FePO_4_	Glucose, phenolic resin, ascorbic acid, starch	∼300 nm	Carbothermal reduction	800°C for 5 h	154.6 mAh g^−1^ (0,1 C)	98.7% (1 C, 20 cycles)	[74]
6	Li_2_CO_3_	FeC_2_O_4_	Gelatin	40–60 nm	Straightforward carbothermal reduction	700°C for 8 h	164.7 mAh g^−1^ (0.3 C)	90.2% (1 C, 500 cycles)	[90]
7	Li_2_CO_3_	FeC_2_O_4_·2H_2_O	Adipic acid	70–250 nm	solid‐state	670°C for 5 h	150 mAh g^−1^ (1 C)	—	[76]
8	Li_2_CO_3_	Fe_2_O_3_	Acetylene black, sucrose	∼150 nm	Carbothermal reduction	600°C for 8 h	155.6 mAh g^−1^ (0.1 C)	100% (0.1 C, 50 cycles)	[58]
9	Li_2_CO_3_	FeSO_4_·7H_2_O	Glucose	0.2–2 µm	Coprecipitation+carbothermal reduction	700°C for 4 h	85.3 mAh g^−1^ (0.5C, ‐20°C)	—	[61]
10	Li_2_CO_3_	FeC_2_O_4_·2H_2_O	—	100–400 nm	solid‐state reaction	600°C for 3 h	160 mAh g^−1^ (0.1 C)	—	[91]
11	Li_2_CO_3_	FePO_4_·2H_2_O	Cotton fiber	50‐100 nm	Template‐assisted	700°C for 6 h	167 mAh g^−1^ (0.2 C)	94.7% (5 C, 100 cycles)	[37]

No.	Li source	Fe source	C source	Particle size	Synthesis method	Sintering condition	Specific capacity	Cycling stability	Refs.
12	LiOH	FePO_4_	Sucrose	∼400 nm	High‐temperature solid‐state carbothermal reduction	750°C for 10 h	128.8 mAh g^−1^ (1 C)	98.75% (5 C, 90 cycles)	[92]
13	LiOH	FeSO_4_	Glucose, rice husk char	80–150 nm	Crystallization, spray drying and rice husk char embedded	700°C for 12 h	158 mAh g^−1^ (0.2 C)	—	[46]
14	LiOH	FeSO_4_·7H_2_O	L‐Lysine	∼30–70 nm	Solvothermal	700°C for 6 h	96 mAh g^−1^ (1 C)	95.06% (1 C, 650 cycles)	[93]
15	LiOH·H_2_O	FePO_4_·2H_2_O	Sucrose	—	Aqueous ball milling, carbon thermal reduction, rheological phase assisted	700°C for 10 h	153 mAh g^−1^ (1 C)	98% (5 C, 200 cycles)	[57]
16	LiOH·H_2_O	FePO_4_·2H_2_O	Glucose	<100 nm	Hydrothermal+carbothermal reduction	600°C for 5 h	163.8 mAh g^−1^ (1 C)	95.8% (1 C, 500 cycles)	[39]
17	LiOH·H_2_O	FeSO_4_·7H_2_O	PAN‐b‐PMMA copolymer	100–200 nm	Carbothermal reduction	700°C for 2 h	165.3 mAh g^−1^ (0.2 C)	98% (0.2 C, 100 cycles)	[85]
18	LiOH·H_2_O	FeSO_4_·7H_2_O	Ascorbic acid	2 µm	Low‐temperature hydrothermal	180°C for 3 h	151 mAh g^−1^ (0.1 C)	∼100% (0.1 C, 30 cycles)	[94]
19	LiOH·H_2_O	FeSO_4_·7H_2_O	Adenosine triphosphate, sucrose	150 nm	Solvothermal	700°C for 6 h	154.27 mAh g^−1^ (1 C)	90.02% (10 C, 100 cycles)	[33]
20	LiH_2_PO_4_	FeC_2_O_4_	Tween60‐Span60	50–90 nm	Solid‐state	700°C for 10 h	166 mAh g^−1^ (0.1 C)	∼100% (0.1 C, 400 cycles)	[78]
21	LiH_2_PO_4_	Fe_2_O_3_	C_6_H_12_O_6_·H_2_O	—	thermal reduction	700°C for 10 h	153.8 mAh g^−1^ (0.1 C)	84% (0.5 C, 1400 cycles)	[65]

No.	Li source	Fe source	C source	Particle size	Synthesis method	Sintering condition	Specific capacity	Cycling stability	Refs.
22	LiH_2_PO_4_	6H_5_FeO_7_	Citric acid, sucrose	18.5 nm	Sol‐gel	700°C for 10 h			[95]
23	Li_3_PO_4_	FeSO_4_·7H_2_O	Sucrose, ascorbic acid	0.5–1 µm	Hydrothermal	650°C for 10 h	119.0 mAh g^−1^ (0.1 C)	∼100% (0.1 C, 100 cycles)	[96]
24	CH_3_COOLi·2H_2_O	Fe(NO_3_)_3_·9H_2_O	Citric acid	100–300 nm	Sol‐gel	700°C for 10 h	150 mAh g^−1^ (0.1 C)	—	[97]
25	CH_3_COOLi·2H_2_O	FeC_2_O_4_·2H_2_O	MCNTs	100–200 nm	Solid‐state	—	145.0 mAh g^−1^ (0.5 C)	—	[98]
26	CH_3_COOLi·2H_2_O	FeC_2_O_4_·2H_2_O	Polyvinyl alcohol	50–100 nm	Spray‐drying+post‐annealing	600°C for 10h	139.4 mAh g^−1^ (0.2 C)	92.4% (10 C, 50cycles)	[99]
27	CH_3_COOLi	FeC_2_O_4_·2H_2_O	—	40–50 nm	Low heating solid‐state coordination+microwave heating	—	125 mAh g^−1^ (C/2)	—	[53]
28	CH_3_COOLi	FeSO_4_·7H_2_O	—	3 µm	Hydrothermal	170°C for 12 h	145 mAh g^−1^ (0.1 C)	—	[100]
29	LiF	FeC_2_O_4_·2H_2_O	—	∼500 nm	Two‐step solid‐state	600°C for 24 h	151 mAh g^−1^ (1/15 C)	—	[47]
30	LiNO_3_	Fe(NO_3_)_3_	Ascorbic acid, sugar		Co‐precipitation	600°C for 16 h	169 mAh g^−1^ (0.1 C)	85% (1 C, 100 cycles)	[101]
31	LiNO_3_	Fe(NO_3_)_3_·9H_2_O	C_2_H_5_NO_2_, CTAB	2–50 nm	Combustion synthesis	700°C for 3 h	146 mAh g^−1^ (0.1 C)	98% (1 C, 50 cycles)	[34]
32	LiH_2_PO_4_, LiOH·H_2_O	FeCl_2_·4H_2_O	Sucrose	1 µm	Spray drying	650°C for 8 h	161.15 mAh g^−1^ (0.1 C)	—	[102]

## 3 Morphology

Morphology is an important factor that can be tailored to control the LFP battery performance. Scanning electron microscopy (SEM) indicates that both the lithium precursor and sintering temperature strongly influence LFP morphology. Using LiF at 600°C yields relatively uniform particles (∼500 nm), which deliver capacities of 151 mAh g^−1^ at 1/15 C and 54.8 mAh g^−1^ at 10 C [47]. However, Li_2_CO_3_ treated at 800°C produces larger particles (>5 µm). Core–shell LFP and LFP /FeS/C composite fibers were prepared by controlling morphology, metallic phase formation, carbon coating, and cation doping. This specific structure improves the electronic conductivity of the LFP cathode [103].

**FIGURE 8:**
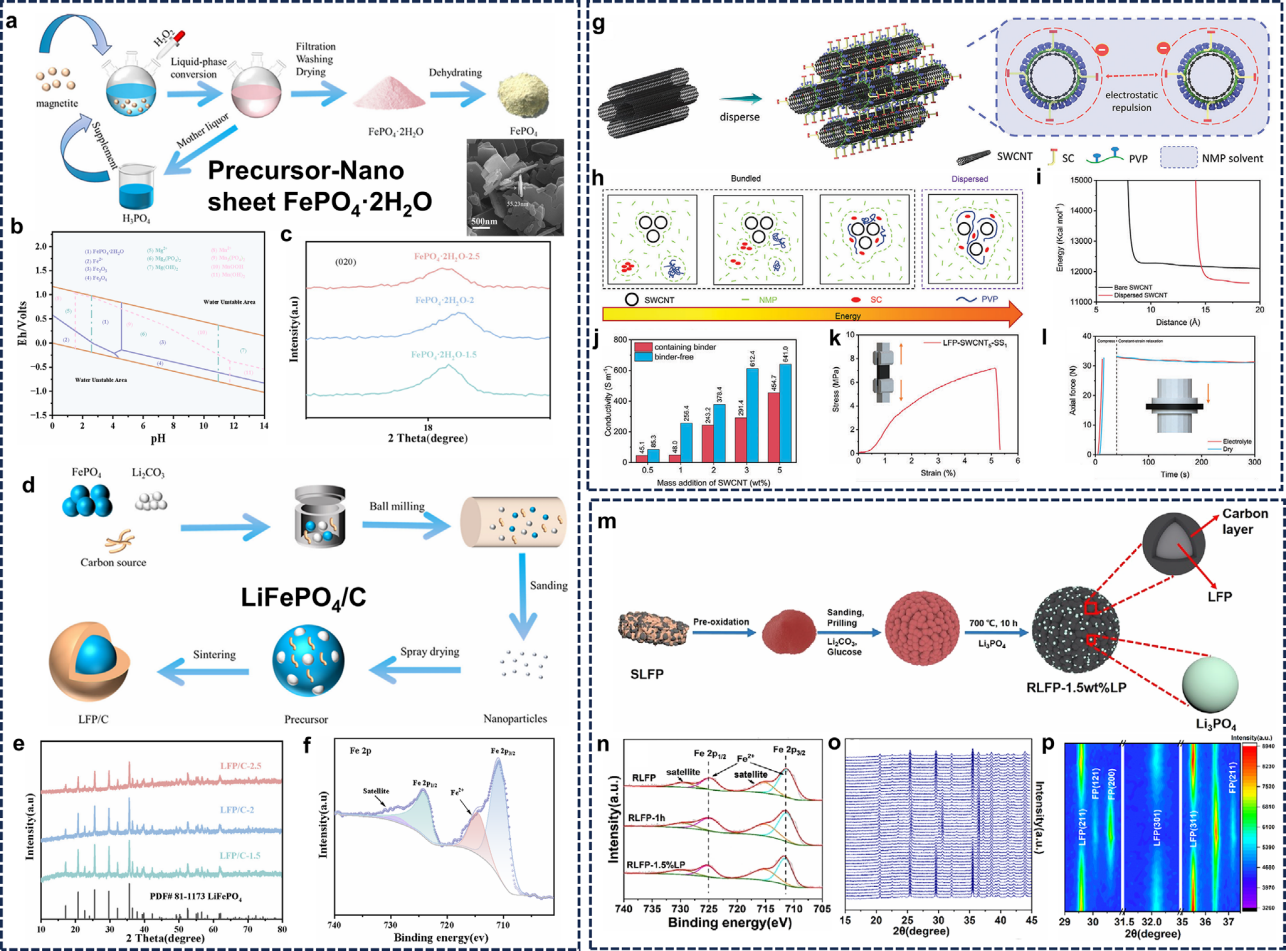
(a–f) Investigation of the nanosheet FePO_4_·2H_2_O on the (020) face by controlling the ratio of phosphate to Fe, reproduced with permission [105]. Copyright 2024, American Chemical Society; (g‐l) Construction of a binder‐free and self‐supporting LiFePO_4_ cathode using mono‐dispersed ultra‐long SWCNTs with enhanced electronic transport and mechanical robustness, reproduced with permission [106]. Copyright 2022, Wiley‐VCH; (m‐p) Spherical micro nano particles shorten the Li^+^ diffusion path and show good electrochemical performance, reproduced with permission [110]. Copyright 2024, American Chemical Society.

2D morphologies, including nanoplatelets and nanosheets [104], offer another route to improving electrochemical performance by exposing large fractions of electrochemically active facets. Thin platelet structures reduce lithium diffusion distances across the shortest dimension while maintaining lateral dimensions sufficient for improved packing. These features enable high specific capacities across a wide range of rates, alongside relatively stable cycling behavior when appropriate surface modifications are applied. FePO_4_·2H_2_O nanosheet precursors derived from low‐cost Fe_3_O_4_ ore were used to synthesize LFP/C and yielded 150.5 mAh g^−1^ at 1 C with >96% capacity retention after 450 cycles at 2 C (Figure 8a–f) [105]. The optimized performance could be due to suppressed crystal growth along the [020] direction by optimizing the phosphate‐to‐iron ratio (P/Fe = 2.5) (Figure 8c). However, the increased surface area of 2D structures may lead to high interfacial degradation. It makes it necessary to optimize electrolytes to ensure long‐term stability.

Single‐walled carbon nanotubes (SWCNTs) have emerged as highly effective conductive additives for LFP cathodes owing to their exceptional electrical conductivity, high aspect ratio, and ability to form interconnected conductive networks at low loading levels (Figure 8g–l) [106]. A scalable dispersion strategy based on electrostatic dipole interactions and steric stabilization enabled the production of well‐dispersed ultra‐long SWCNTs that effectively interconnected LiFePO_4_ particles and maintained continuous electron‐transport pathways with only 0.5 wt.% additive content. The resulting self‐supporting, binder‐free LFP/SWCNT electrodes exhibited a high electrical conductivity of 1197 S m^−1^ (Figure 8j). The excellent mechanical robustness at 7.2 MPa stress (Figure 8k) and 5% strain enables high mass loading and efficient charge transport. The as‐prepared cathodes delivered capacities of 161.5 mAh g^−1^ at 0.5 C and 130.2 mAh g^−1^ at 5 C, while retaining 87.4% of their capacity after 200 cycles at 2 C.

Some specific LFP morphologies show remarkably high electrochemical performance. 3D hierarchical LiFePO_4_ /C microspheres were synthesized by a facile solvothermal process in a solution of glycerol and water with the addition of citric acid, and the LFP/C materials exhibit a superior discharge capacity of 162 mAh g^−1^ at 0.1 C [107]. A higher capacity of 163.9 mAh g^−1^ was reported for a similar structure [108]. In these structures, nanoparticles facilitate rapid lithium‐ion transport, and the microscale secondary phases enable high tap density. Therefore, excellent capacities are often obtained. A spherical LFP cathode material was prepared using a controlled crystallization and carbothermal reduction method, with FePO_4_·xH_2_O as the iron precursor and Li_2_CO_3_ and glucose as raw materials [109]. The experimental results show that this synthetic material has a compaction density of 1.8 g/cm^3^ and a volumetric energy density of 233.5 mAh cm^−3^, which is significantly higher than those reported for non‐spherical LFP materials. It was also deemed that the spherical micronano particle has a short Li diffusion path. Therefore, it shows a low charge‐transfer resistance (Figure 8m–p) [110]. In situ XRD showed the coexistence of LiFePO_4_ (LFP) and FePO_4_ (FP) phases during charging. FP phase gradually forms as Li^+^ ions are extracted from the olivine lattice. Upon full charge, the disappearance of the characteristic LFP reflections signifies the complete transformation to FP. During discharge, Li^+^ ions are reinserted into the structure, resulting in the reappearance and coexistence of both LFP and FP phases as the reverse phase transformation proceeds (Figure 8o,p).

Porous and hollow morphologies further exemplify the importance of structural design in mitigating degradation mechanisms. The optimized LFP/carbon materials derived from mesoporous iron phosphate precursors deliver an initial discharge capacity of 159.29 mAh g^−1^ at 0.1 C (a 27.10% increase) and a markedly reduced charge‐transfer resistance of 48.32 Ω (an 88.16% decrease), as compared to the cathode with non‐porous precursors [89].

The morphological design has to balance nanoscale and microscale regimes, as the nanoscale is essential for fast kinetics, while the microscale is desirable for high volumetric energy density [111]. These different morphologies also affect the cycle life. The desired structure should remain stable throughout the lithium insertion and extraction cycle without structural degradation. Further integration of the synthesis method with precursor selection is necessary.

## 4 Doping Elements

Doping is an effective strategy for overcoming LFP's weaknesses as an LIB cathode. Depending on ionic radius, valence state, and chemical compatibility, dopants can be incorporated into the lithium, iron, or polyanion sublattices [24]. The doping influences lattice parameters, defect chemistry, and electronic structure. In this case, lithium‐ion migration pathways will be affected. Subsequently, the electrochemical performance of the doped LFP differs. Although the specific mechanisms differ among doping sites, a common objective is to improve charge transport while preserving the structural stability that underpins the long cycle life of LFP.

### 4.1 Li‐Site Doping

The Li sublattice directly forms the 1D diffusion channels responsible for lithium‐ion transport in olivine LiFePO_4_. Consequently, substitution at the Li site primarily affects Li^+^ mobility through lattice expansion, defect generation, and diffusion‐channel modification. Alkali metal ions, such as sodium and potassium, have been doped at the Li site [112]. The radii of these ions are slightly larger than those of Li ions, and the lattice is expanded, which facilitates Li^+^ transport. Cho et al. [113] doped Na into the Li site and found that 3% Na doping enhanced Li^+^ diffusivity and delivered a better initial discharge capacity of 151.9 mAh g^−1^, which is much better than 129.9 mAh g^−1^ for the non‐doped LFP battery. The improvement was attributed to enlarged Li‐ion migration pathways and reduced diffusion resistance.

### 4.2 Fe‐Site Doping

The FeO_6_ octahedral framework governs both electronic transport and electrochemical redox behavior in LFP. As a result, Fe‐site substitution has become the most extensively investigated doping strategy. Transition‐metal dopants such as La, Ti, Nb, Mg, Cu, and In primarily function by modifying the electronic structure, regulating defect chemistry, and reducing charge‐transfer resistance [114]. These dopants improve the intrinsic electronic conductivity by narrowing the bandgap, and simultaneously bolster thermal stability [23, 26, 115].

**FIGURE 9:**
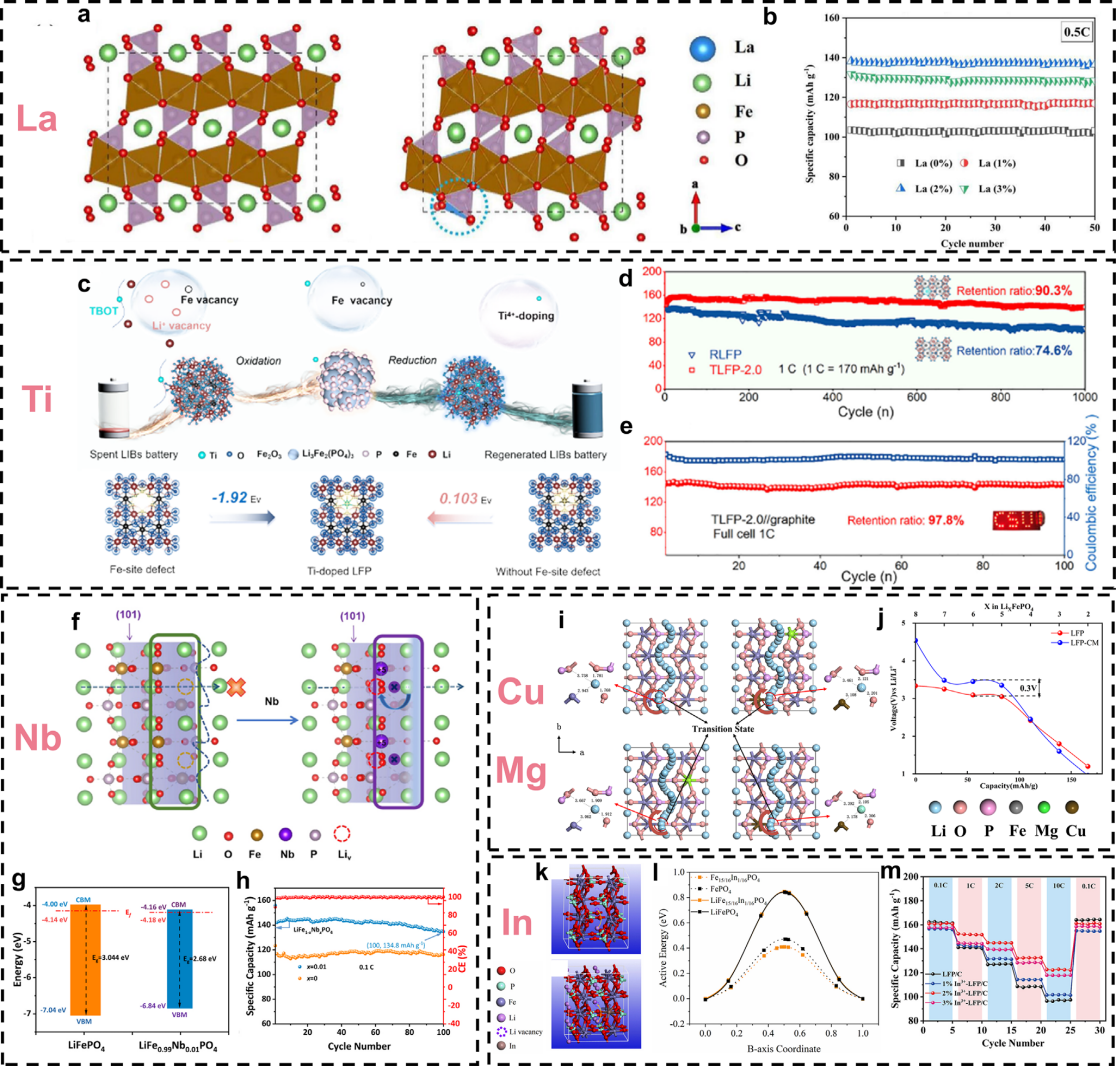
Doping strategies for LFP. (a,b) An optimization of lanthanum doping for LFP, reproduced with permission [116]. Copyright 2024, Springer Nature; (c–e) Illustration of the diagram of Ti‐doped LFP and electrochemical performance, reproduced with permission [118]. Copyright 2026, Wiley‐VCH; (f–h) Mechanism and performance of Nb doping in LiFePO_4_ cathodes, reproduced with permission [119]. Copyright 2025, Elsevier; (i,j)reproduced with permission [122]. Copyright 2025, Elsevier; (k–m) In‐doped LFP exhibits enhanced diffusion energy barriers, reproduced with permission [123]. Copyright 2023, Elsevier.

Lanthanum incorporation has been shown to enhance both lithium‐ion transport and electronic conductivity in LFP/C. As a result, La^3+^‐modified samples exhibit improved electrochemical performance, delivering 119.21 mAh g^−1^ at 5 C after 100 cycles, compared with 83.21 mAh g^−1^ for the undoped material. This improvement is accompanied by a marked increase in electronic conductivity of 4.4 × 10^−3^ S cm^−1^, which is higher than 2.78 × 10^−6^ S cm^−1^ for non‐doped LFP [114]. The lithium diffusion coefficient is 5.68 × 10^−14^ cm^2^ s^−1^ vs. 9.89 × 10^−15^ cm^2^ s^−1^. Another study showed that a 2% La replacement was optimal within the 1%–3% range (Figure 9a,b) [116]. DFT calculations show that incorporating 2% La into LiFePO_4_ reduces the band gap from 3.44 to 3.27 eV, and both the Li─O and Fe─O bond lengths increase. The longer Li─O bonds indicate weaker interactions between Li and O, which helps to lower the resistance to Li^+^ diffusion. At the same time, the slight increase in Fe─O bond length reduces bond strength and slightly widens Li^+^ migration pathways, facilitating ion transport.

Ti‐ion incorporation has been demonstrated to improve the electrochemical performance of LiFePO_4_ [117]. However, Ti doping must be carefully controlled, as the similar chemical environments of Fe and Li sites often lead to uncontrolled co‐occupation during doping. Zhang et al. [118] first filled the Li site in a spent LFP, and then Ti could occupy the Fe site. They found that the optimized Ti content was 2% and that the best capacity was 155 mAh g^−1^ at 1.0 C. The cathode retained 90.3% capacity after 1000 cycles (Figure 9c,d).

Nb doping represents a promising strategy for electronic‐structure engineering. By introducing high‐valent Nb^5+^ ions, Fe^2+^ is partially oxidized into Fe^3+^. This facilitates electron delocalization through Fe─O─Nb orbital hybridization. This d‐p‐d coupling reduces the band gap and lowers the polaron migration barriers, which improves charge‐transfer kinetics. Simultaneously, Nb incorporation facilitates Li^+^ diffusion by expanding transport channels and reducing diffusion resistance. These synergistic effects lead to improved rate performance and cycling stability, demonstrating the potential of orbital‐level design in overcoming the intrinsic kinetic limitations of LFP cathodes (Figure 9f–h) [119].

Magnesium doping has also been extensively investigated owing to the comparable ionic radii of Fe^2+^ and Mg^2+^. MgO, MgSO_4_, and MgHPO_4_ have been chosen as the magnesium source for doping LFP. No effect on the structure and crystallinity was found. Increasing Mg content shifts diffraction peaks to higher angles because smaller Mg^2+^ ions substitute for Fe or Li ions. The introduction of Mg^2+^ can create lithium vacancies to compensate for the charge imbalance, thereby increasing lithium‐ion mobility and enhancing ionic conductivity. It was shown that 1% MgHPO_4_–LFP exhibited enhanced cycling stability with ∼101% capacity retention after 30 cycles at 0.5 C, which may be attributed to more accessible Li^+^ transport pathways [120]. However, excessive Mg incorporation narrows Li^+^ diffusion channels, hindering Li‐ion intercalation/extraction [121]. Therefore, it is necessary to control dopant concentration to balance performance gains. To further improve transport kinetics, Cu and Mg have been explored. It is shown that co‐doping reduces the bandgap and lowers the Li^+^ diffusion barrier. Subsequently, the co‐doped LFP has improved electronic conductivity and lithium‐ion mobility. Mg‐Cu co‐doping not only improves charge‐transport kinetics but also elevates the operating voltage to 3.64 V, enabling higher energy output while maintaining a theoretical capacity of approximately 170 mAh g^−1^, higher than 165 mAh g^−1^ for pristine LFP (Figure 9i,j) [122].

Indium doping has been shown to be another effective approach to regulate the transport properties of LFP. A 2% In^3^^+^ was doped into LFP. 160 mAh g^−1^ was achieved, and the capacity was maintained at 98% over 700 cycles (Figure 9k–m) [123]. DFT calculations indicated that LiFe_1‐x_In_x_PO_4_ introduces impurity states near the Fermi level that enhance electronic conductivity. The synergistic enhancement of Li^+^ transport and charge‐transfer kinetics was further verified by the improved high‐rate performance (5–10 C). Particular attention should be given to the structural stability, as indium has a high valence state and a large crystal size.

Although the doping strategy has been proven effective, the continued development of doping strategies is still needed. Particular attention should be given to combining advanced morphological design with scalable synthesis methods to achieve a desirable LFP cathode for industrial applications [124].

### 4.3 Polyanion and Anion‐Site Doping

Unlike Li‐ and Fe‐site dopants, substitutions within the PO_4_ polyanion framework primarily modify the local inductive effect and metal–oxygen bonding characteristics. Substituting the polyanion site with metalloids B, Si, or the O site with electronegative species fluorine (F^−^) or sulfur (S^2−^) reconfigures the local inductive effect [24, 25]. This shift in the metal‐oxygen covalent character optimizes the redox potential and enhances interfacial electrochemical stability [125, 126]. Among these approaches, sulfur doping, typically achieved by partial substitution of oxygen or phosphate groups, has been widely explored to modify the polyanion framework and enhance electronic conductivity. The substitution of oxygen by sulfur increases covalency within the lattice and narrows the bandgap, thereby facilitating electron transport. Sulfur doping increases covalency and narrows the bandgap. For example, DFT calculations on a 2 × 2 × 1 supercell show that substituting three O atoms with S (compositionally equivalent to LiFePO_3.625_S_0.375_) reduces the bandgap from 3.58 to 0.31 eV [127]. Such electronic‐structure modification improves conductivity and enhances rate performance.

The driving forces behind LFP development are its safety, durability, and long cycle life. Its practical application was limited by its intrinsically low electronic conductivity and slow Li diffusion. To bridge the gap between theoretical potential and practical performance, extensive efforts have focused on multidimensional material optimization. Recent advances show that precursor selection and morphological control are critical and can be incorporated into the synthesis method [85].

**FIGURE 10:**
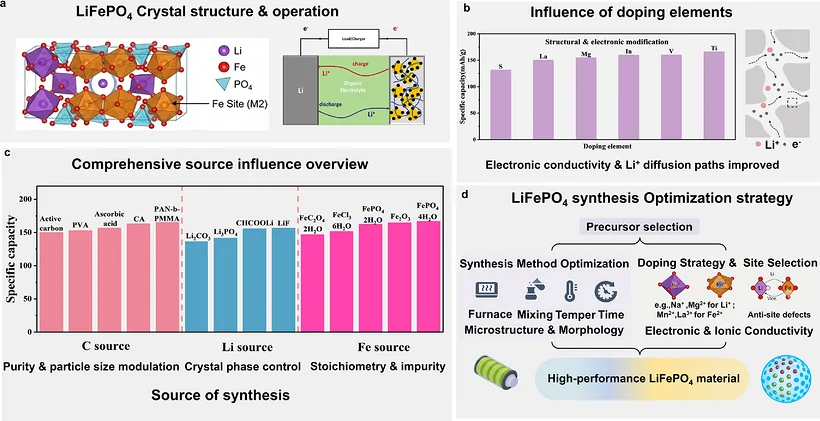
(a) Illustration of the LFP structure and its battery; (b)Influence of doping elements on the capacity of the LFP batteries [116, 118, 123, 128, 130, 132]; (c) Influence of sources on the capacity of the LFP batteries [60, 66, 69, 83, 85, 134, 135, 136, 137, 138, 141, 142, 143, 145, 147, 149]; (d) Optimization strategy for LFP batteries.

In this work, we present a comprehensive overview of some optimization strategies. We outline a unified roadmap for engineering high‐performance LFP materials with improved electrochemical performance by identifying the correlations among synthesis methods, dopants, and resulting microstructural properties. The olivine structure defines the arrangement of Li, Fe, and PO_4_ units, which governs Li^+^ insertion and extraction during battery operation (Figure 10a). To overcome the inherent electronic limitations of pristine LFP, targeted elemental doping (utilizing elements such as S, La, Mg, In, V, and Ti) (Figure 10b) is shown to systematically increase specific capacity (approaching 150–170 mAh g^−1^) [128, 129, 130, 131, 132, 133]. This improvement is driven by structural and electronic modifications that simultaneously widen Li^+^ diffusion pathways and enhance bulk electronic conductivity. The electrochemical performance is highly sensitive to the selection of the precursor (Figure 10c). The choice of carbon source (e.g., active carbon, PVA) significantly affects capacity due to enhanced conductivity. While the oxidation state of iron and solubility play a critical role in the morphology and, as a consequence, in the rate performance and cyclability. It requires a synergistic optimization strategy to achieve high‐performance LFP [54, 134, 135, 136, 137, 138, 139, 140, 141, 142, 143, 144, 145, 146, 147, 148, 149]. A high‐performance LFP cathode can be obtained by precisely controlling the synthesis method with various parameters, occupying the right sites with different dopants (e.g., substituting Na^+^ or Mg^2+^ for Li at Li sites; Ti at Fe sites), or tailoring particular morphologies (Figure 10d).

## 5 Conclusion and Perspectives

The new energy vehicle market has provided more opportunities for the future development of lithium‐ion batteries. The advantages of high safety, high cycle stability, and low environmental impact of lithium iron phosphate cathode materials make it the first choice for power batteries. Improving the energy density and power density of lithium batteries is the only way to expand their application range. Industry and academia should make it their goal to discuss it in depth. LiFePO_4_ (LFP) has been widely investigated at both the scientific and industrial scales to reduce dependence on cobalt supplies due to economic and environmental concerns. More attention should be given to the approaches used to design and synthesize LFP materials to overcome the intrinsic kinetic bottlenecks of its low electronic conductivity and sluggish 1D lithium‐ion diffusion.

In this review, we summarize recent advances in the design and preparation of LFP nanomaterials, highlighting how precursor chemistry, carbon incorporation, particle morphology, and targeted doping collectively govern phase formation, electronic and ionic transport, and electrochemical performance. LFP synthesized using different precursor systems exhibits distinct particle sizes, morphologies, and surface characteristics, all of which strongly influence battery performance. Nanomaterials with tailored architectures have played an important role in enhancing the charge–discharge characteristics of lithium‐ion battery applications. The effect of Li, Fe, and C sources on the preparation of lithium iron phosphate was systematically discussed, demonstrating that precursor selection has a significant influence on crystallinity, particle growth, carbon distribution, and ultimately electrochemical behavior. Furthermore, doping elements, such as sulfur, lanthanum, titanium, niobium, magnesium, and indium, have been shown to improve capacity, cyclability, and rate performance by modifying the electronic structure and lithium‐ion transport pathways. These findings demonstrate that performance optimization requires a synergistic combination of synthesis strategy, precursor selection, and post‐synthetic modification. Establishing clear structure‐synthesis‐performance relationships remains critical for translating laboratory‐scale advances into viable industrial‐scale technologies.

Future advances in LFP cathodes will increasingly be driven by the transition from laboratory‐scale materials optimization to manufacturing‐oriented design. Industrial leaders such as Contemporary Amperex Technology Co., Limited (CATL), BYD Company Limited (BYD), EVE Energy Co., Ltd. (EVE Energy), and Gotion High‐tech Co., Ltd (Gotion High‐tech) have demonstrated that breakthroughs in LFP technology result not only from materials discovery but also from advances in process engineering, supply‐chain integration, and large factory‐scale manufacturing. Further refinement of scalable and reproducible synthesis routes is required to balance materials uniformity with reduced energy consumption and environmental impact. This could be achieved through sustainable precursors, low‐temperature processes, and continuous manufacturing approaches suitable for industrial‐scale production. For example, the selective recovery of battery‐grade lithium salts and iron oxides from spent LFP black mass via low‐temperature NaOH roasting provides a viable route for integrating recycled materials into industrial precursor supply chains. In contrast to nickel‐rich cathodes, LFP recycling primarily benefits from direct cathode regeneration and material reuse rather than breaking the battery down into base metals with acid. By replenishing lithium and reconstructing the olivine framework, degraded LFP can be regenerated to near‐battery‐grade quality while significantly reducing energy consumption, chemical usage, and processing costs. This is more favorable in large‐scale recycling and regeneration and is widely accepted by CATL, BYD, EVE Energy, and Gotion High‐tech.

Although carbon remains essential in LFP batteries, future research should prioritize reducing inactive components and increasing tape density and fast‐charging ability while maintaining efficient transport kinetics. In this context, hierarchical conductive networks based on hybrid 1D and 2D carbon frameworks (e.g., CNT–graphene assemblies) are expected to maintain electronic percolation under high compaction. Electrode‐level density‐gradient architectures, combining dense sublayers near the current collector with more porous ion‐accessible surfaces, further help reconcile volumetric energy density with rate capability. From a processing perspective, controlled sintering and post‐treatment strategies that minimize internal voids, together with optimized carbon coatings, can enhance skeletal density without compromising electrochemical accessibility. Collectively, these approaches indicate that achieving tap densities above ∼2.0 g cm^−3^ is feasible through integrated control of particle architecture, conductive network design, and electrode‐level structuring, rather than single‐parameter optimization. Another demand is that fast‐charging electric vehicles require cathodes capable of delivering more than 120 mAh g^−1^ at 10 C while maintaining long‐term cycling stability, with practical charging targets approaching 80% state‐of‐charge within 10–15 min. For stationary energy‐storage applications, cycle lifetimes exceeding 10 000 cycles will become increasingly important.

Operando characterization and data‐driven modelling are expected to accelerate the optimization of dopant selection, defect engineering, and interfacial design. By combining high‐throughput computational screening, machine‐learning potentials, and advanced foundation models for defect analysis, researchers can accelerate the discovery of optimal dopants, defect chemistries, and interfacial architectures. Some co‐doping, e.g., Sm and Gd, Cu, and Mg, La and Y, have been investigated. AI‐assisted materials selection will optimize co‐doping elements; for example, combining Lanthanides could be an optimal choice. Regarding the application of LFP in solid‐state batteries, an artificial buffer layer or a polymer interface could improve the interface. For example, Li_3_VO_4_ and NASICON‐type Li_1.3_Al_0.3_Ti_1.7_(PO4)_3_ (LATP) can serve as fast‐ion‐conducting bridges to reduce interfacial impedance, with their effectiveness strongly dependent on nanoscale thickness control (∼2.5–5 nm) to balance protection and Li^+^ transport. Polymer–ceramic composite electrolytes (e.g., PEO‐based systems with ceramic fillers) are another choice. It provides mechanically compliant interfaces that accommodate volume changes while improving macroscopic contact stability. Advanced processing strategies, including in situ polymerization infiltration and atomic layer deposition, further enable conformal interlayers and uniform ion flux across porous cathodes.

Industrial experience reveals that performance under extreme conditions remains hindered, as low‐temperature operations and fast charging are limited by slow lithium‐ion diffusion and polarization. This ongoing challenge has prompted companies to invest in innovative strategies, including single‐crystal LFP, hierarchical microsphere architectures, advanced conductive frameworks, and new electrolyte materials. For example, a weakly solvating ether (WSE) electrolyte based on 2‐methyl‐tetrahydrofuran has been proven to be a good material for an LFP‐based battery operating at −30°C for 1600 h. Additional cycles should be tested, and fast‐charging conditions (10 C or 20 C) should be further investigated.

In summary, future progress in LFP technology will rely on the integration of materials innovation, scalable manufacturing, and circular economy principles. Bridging the divide between laboratory‐scale discoveries and industrial application will be essential to fully realize the potential of LFP as a safe, sustainable, and cost‐effective cathode material for next‐generation energy storage systems.

## Author Contributions

**Lihui Yin**: conceptualization, writing – original draft, writing – review & editing. **Yao Jiang**: formal analysis, software, data curation. **Jun Wang**: formal analysis, writing – original draft. **Bin Li**: data curation, software. **Feng Liu**: formal analysis, software. **Cairong Jiang**: conceptualization, funding acquisition, writing – review & editing. **Yali Yao**: writing – review & editing. **Jianwu Wen**: data curation, formal analysis. **Chami N.K. Patabendige**: writing – review & editing. **Venkataraman Thangadurai**: writing – review & editing. **Jianjun Ma**: funding acquisition, supervision, writing – review & editing.

## Conflicts of Interest

The authors declare no conflicts of interest.

## Data Availability

The data that support the findings of this study are available from the corresponding author upon reasonable request.
